# Investigating the Association Between Telemedicine Use and Timely Follow-Up Care After Acute Cardiovascular Hospital Encounters

**DOI:** 10.1016/j.jacadv.2022.100156

**Published:** 2022-12-30

**Authors:** Mitchell Tang, A Jay Holmgren, Erin E. McElrath, Ankeet S. Bhatt, Anubodh S. Varshney, Simin G. Lee, Muthiah Vaduganathan, Dale S. Adler, Robert S. Huckman

**Affiliations:** a Harvard University, Cambridge, Massachusetts, USA; b Harvard Business School, Boston, Massachusetts, USA; c Department of Medicine and Center for Clinical Informatics and Improvement Research, University of California-San Francisco, San Francisco, California, USA; d Department of Medicine, Brigham and Women’s Hospital, Boston, Massachusetts, USA; e Division of Cardiovascular Medicine, Department of Medicine, Brigham and Women’s Hospital and Harvard Medical School, Boston, Massachusetts, USA; f Kaiser Permanente Division of Research, Oakland, California, USA; g Division of Cardiovascular Medicine, Stanford University, Palo Alto, California, USA; h National Bureau of Economic Research, Cambridge, Massachusetts, USA

**Keywords:** heart failure, post-hospitalization follow-up, readmissions, telemedicine

## Abstract

**Background:**

Telemedicine use increased dramatically during the COVID-19 pandemic; however, questions remain as to how telemedicine use impacts care.

**Objectives:**

The purpose of this study was to examine the association of increased telemedicine use on rates of timely follow-up and unplanned readmission after acute cardiovascular hospital encounters.

**Methods:**

We examined hospital encounters for acute coronary syndrome, arrhythmia disorders, heart failure (HF), and valvular heart disease from a large U.S., multisite, integrated academic health system among patients with established cardiovascular care within the system. We evaluated 14-day postdischarge follow-up and 30-day all-cause unplanned readmission rates for encounters from the pandemic “steady state” period from May 24, 2020 through December 31, 2020, when telemedicine use was high and compared them to those of encounters from the week-matched period in 2019 (May 26, 2019, through December 31, 2019), adjusting for patient and encounter characteristics.

**Results:**

The study population included 6,026 hospital encounters. In the pandemic steady-state period, 40% of follow-ups after these encounters were conducted via telemedicine vs 0% during the week-matched period in 2019. Overall, 14-day follow-up rates increased from 41.7% to 44.9% (adjusted difference: +2.0 percentage points [pp], 95% CI: −1.1 to +5.1 pp, *P* = 0.20). HF encounters experienced the largest improvement from 50.1% to 55.5% (adjusted difference: +6.5 pp, 95% CI: +0.5 to +12.4 pp, *P* = 0.03). Overall 30-day all-cause unplanned readmission rates fell slightly, from 18.3% to 16.9% (adjusted difference −1.6 pp; 95% CI: −4.0 to +0.8 pp, *P* = 0.20).

**Conclusions:**

Increased telemedicine use during the COVID-19 pandemic was associated with earlier follow-ups, particularly after HF encounters. Readmission rates did not increase, suggesting that the shift to telemedicine did not compromise care quality.

During the initial months of the COVID-19 pandemic, the use of telemedicine increased dramatically in the United States due to government shelter-in-place orders, concerns of COVID-19 exposure for both patients and clinicians, and increased reimbursement from payers for telemedicine visits.^1^^,^^2^ Although the use of telemedicine has fallen since the early stages of the pandemic, it has continued to far exceed prepandemic levels. Telemedicine is expected to continue to play an important role alongside in-person care for the foreseeable future. However, the impact of increased telemedicine availability is unclear.

Telemedicine undoubtedly has the potential to improve access and convenience for many patients by reducing the substantial time and cost burdens associated with travel to in-person appointments.^3^, ^4^, ^5^, ^6^ It may also increase clinician efficiency and lower the costs of care by reducing patient no-show rates and overhead costs associated with facility space, equipment, and front desk staff.^5^^,^^7^ There may be other incidental benefits of telemedicine, such as speaking with patients in the comfort of their home or being able to see labeled containers for the medications they are taking.^8^ Conversely, telemedicine may be an imperfect substitute for in-person clinical evaluation due to the lack of physical examination and verified physiological measurements. Other aspects of care such as ordering lab tests or medications may differ in telemedicine vs in-person visits.^9^^,^^10^ Lastly, patients—particularly those with limited financial means—may face barriers to accessing care via telemedicine due to factors such as lack of device access, broadband access, or low digital literacy.^11^, ^12^, ^13^ The magnitudes of these effects are likely context specific, suggesting that maximizing the value of telemedicine requires identifying the right situations for its use.

Outpatient follow-up care after acute cardiovascular hospital encounters may be 1 opportunity to leverage telemedicine’s access and convenience benefits. The weeks following discharge are marked by significant risk of rehospitalization and other adverse outcomes and represents a complex period in which significant changes may be made to patients care plans.^14^, ^15^, ^16^, ^17^ Evidence has shown that timely follow-ups can help support patients' adherence to care plans and reduce the risk of readmission.^18^, ^19^, ^20^, ^21^ Telemedicine offers the potential for increasing patient access to timely follow-ups. On the other hand, physical exams and lab tests are often key components of follow-up visits,^22^^,^^23^ creating reasonable concerns about care quality when telemedicine is used.

In this study, we use data from the Mass General Brigham (MGB) health system to investigate the association of increased telemedicine use during the COVID-19 pandemic with rates of timely follow-up after acute cardiovascular hospital encounters and with the quality of care as measured by readmission rates.

## Methods

### Data

Our study data were pulled from the Epic Systems electronic health record for MGB, a multisite, integrated academic health system in Massachusetts. The data provided records of all hospitalizations, emergency department (ED) visits, observation stays, and outpatient visits across all MGB sites. Because our study was a retrospective analysis of routinely collected data, the Harvard University Area and MGB institutional review boards deemed the study exempt.

### Study population

Our study population included encounters for acute coronary syndrome (ACS), arrhythmia disorders (ADs), heart failure (HF), and valvular heart disease (VHD) resulting in hospitalizations, ED visits, or observation stays discharged from January 1, 2019, to December 31, 2020. Encounter diagnoses were identified by the primary discharge diagnosis according to the International Classification of Diseases-10th Revision coding (Supplemental Table 1). Encounters were only included in our sample if they represented index encounters. In cases where patients had multiple encounters within a 30-day period, including those for noncardiovascular diagnoses, we considered the earliest as the index encounter.

Our study population was further restricted to encounters for patients with established cardiovascular care at MGB, defined as having at least 1 cardiology outpatient visit in the 24 months prior to the index encounter. Restricting to patients with established cardiovascular care at MGB increased the likelihood that follow-ups would also be within system, mitigating the risk of unrecorded follow-ups. Finally, we restricted the study population to encounters in which the patient was discharged to home, as patients discharged to post–acute care facilities or hospice may have context-specific follow-up plans.

### Time period definitions

Encounters were grouped into time periods based on discharge date. In 2020, 3 periods were defined: a prepandemic period from January 1, 2020, to February 29, 2020, a transition period from March 1, 2020, to May 23, 2020, and a COVID-19 pandemic “steady-state” period from May 24, 2020, through December 31, 2020. The week starting on March 15, 2020, which saw significant reductions in MGB outpatient volumes and the first state shelter-in-place order in the United States on March 19, 2020, (California),^24^ was used as a proxy for the onset of the pandemic. The end of our prepandemic period was set 2 weeks prior to March 15, 2020, so that no prepandemic encounter had a 14-day postdischarge window overlapping with the pandemic.

The transition period represents the early months of the pandemic during which time outpatient care at MGB was significantly disrupted; in-person care was significantly curtailed; and video telemedicine had not been broadly adopted (Supplemental Figures 1 and 2). The system-wide reopening for the MGB health system occurred on May 18, 2020; the transition period end date of May 23, 2020, represents the last day in the week including May 18, 2020.

During the pandemic steady-state period, total outpatient visit volumes rebounded to prepandemic levels, but telemedicine visits continued to play a substantial role in outpatient care delivery. During this period, video visits supplanted audio-only visits as the primary form of telemedicine visit. This change was supported in part by technological improvements that enabled easier provider and patient use of video visits. These included streamlining the process to initiate video visits within Epic and implementation of Doximity’s “Dialer Video”, which enabled patients to join secure video visits through a texted link without any additional application downloads or account creation. However, during this period, audio-only visits continued to represent a meaningful portion of telemedicine visits, due to bandwidth constraints and technology preferences among patients.

To control for seasonal effects, 3 analogous week-matched periods were identified in 2019: a prepandemic analog from January 1, 2019, to March 2, 2019; a transition analog from March 3, 2019, to May 25, 2019; and a pandemic steady state analog from May 26, 2019, to December 31, 2019.

### Measures

Our primary exposure of interest was increased outpatient telemedicine use in the 2020 pandemic steady-state period compared with the week-matched period in 2019. Our primary outcome measure was the proportion of encounters with a follow-up within 14 days of discharge. A follow-up was defined as a completed outpatient visit with a cardiovascular, cardiac surgery, or general internal medicine physician or advanced practice provider. Fourteen days was selected to be consistent with HF guidelines, which recommend follow-up within 7 to 14 days after discharge, and prior literature.^18^^,^^25^^,^^26^ Encounter follow-ups were categorized by the most intensive modality used within the 14 days after discharge, with audio-only visits being the least intensive, in-person the most intensive, and video visits lying in between. Sensitivity analyses were conducted using 7-day, 21-day, and 28-day thresholds for timely follow-up. We also examined days to first follow-up for encounters with at least 1 follow-up within 60 days of discharge.

As a preliminary investigation of potential associations between telemedicine use and the quality of follow-up care, we also examined the 30-day all-cause unplanned readmission rate. Readmission included any unplanned hospitalization, ED visit, or observation stay regardless of the primary diagnosis code within 30 days of the index encounter discharge.

### Statistical analysis

Our primary statistical comparisons were mean 14-day follow-up rates and readmission rates for encounters from the pandemic steady-state period in 2020 against those from the analogous period in 2019. Comparisons were also included for the prepandemic and transition periods to detect potential 2020 vs 2019 pretrends and the effect of the pandemic onset, respectively. As a sensitivity analysis, we also conducted 2020 vs 2019 comparisons by month rather than our 3 aggregated periods.

Comparisons were conducted across the pooled set of acute cardiovascular encounters and separately for each of the 4 conditions. We examined both unadjusted and adjusted comparisons. Unadjusted comparisons used 2-sample *t*-tests. Adjusted comparisons used ordinary least squares regression with controls for encounter characteristics, such as type of encounter (hospitalization, ED, or observation); length of stay; whether the patient was discharged with home health services; whether the encounter included an intensive care unit (ICU) stay; whether the encounter was unplanned; and patient characteristics, such as sex, age at discharge, and the patient’s number of MGB outpatient cardiology visits in the year prior to the encounter. Pooled comparisons included additional indicator variables for the 4 conditions studied. All analyses were conducted using R (v4.0.2), with 2-sided *P* values and heteroskedasticity robust standard errors.

## Results

### Study cohort characteristics

Our final sample included 6,026 encounters discharged between January 1, 2019, and December 31, 2020. Counts of encounters passing each inclusion criteria are provided in Supplemental Table 2. In 2020, there were 2,684 encounters: 490 during the prepandemic period, 421 during the transition period, and 1,773 during the pandemic steady state period. In 2019, there were 3,342 encounters: 570 during the prepandemic-matched period, 747 during the transition-matched period, and 2,025 during the pandemic steady-state-matched period (Table 1). Across all periods, the proportion of encounters due to each primary diagnosis was as follows: 18% for ACS, 37% for AD, 30% for HF, and 15% for VHD. Encounter volumes were lower in all 3 2020 periods relative to 2019. The 2020 transition period also had a lower average patient age (68.9 vs 70.9, *P* = 0.02), higher proportion of unplanned encounters (80% vs 74%, *P* = 0.01), and higher proportion of encounters requiring an ICU stay (27% vs 21%, *P* = 0.03) than those from the week-matched 2019 period. In the 2020 pandemic steady state, we also observed a higher proportion of encounters requiring an ICU stay (29% vs 19%, *P* = <0.01), a lower average number of MGB cardiology visits in the year prior to the encounter (4.49 vs 4.80, *P* = 0.03), and a lower proportion of HF encounters and higher proportion of AD encounters (primary diagnosis mix *P* = 0.04) relative to the week-matched 2019 period. Patient-reported race and ethnicity were not uniformly collected across our sample and, thus, were not examined.

**Table 1:** Study Cohort Encounter Characteristics

		Prepandemic			Transition			Pandemic Steady State		
		2019	2020	2020 vs 2019 ***P*Value**	2019	2020	2020 vs 2019 ***P*Value**	2019	2020	2020 vs 2019 ***P*Value**
Date ranges	Start date	1/1/19	1/1/20	-	3/3/19	3/1/20	-	5/26/19	5/24/20	-
	End date	3/2/19	2/29/20	-	5/25/19	5/23/20	-	12/31/19	12/31/20	-
	# of weeks	9	9	-	12	12	-	32	32	-
Encounter volumes	# of encounters	570	490	-	747	421	-	2,025	1,773	-
	# of encounter/week	63.3	54.4	-	62.3	35.1	-	63.3	55.4	-
Encounter type	% Inpatient	84%	80%	0.04	82%	81%	0.58	81%	82%	0.16
	% ED	11%	12%		12%	11%		13%	11%	
	% Observation	5%	9%		7%	8%		6%	7%	
Patient demographics	Average age	69.9	70.4	0.56	70.9	68.9	0.02	70.1	70.9	0.07
	% from rural zip	1.1%	0.8%	0.69	1.2%	0.7%	0.39	1.2%	1.0%	0.52
	% female	39%	40%	0.76	42%	44%	0.53	39%	42%	0.07
	Average median income from patient zip	$92,032	$91,067	0.65	$91,046	$92,735	0.44	$91,201	$91,231	0.98
Primary diagnosis	% ACS	17%	20%	0.57	17%	17%	0.23	18%	19%	0.04
	% AD	35%	36%		32%	37%		37%	40%	
	% HF	33%	31%		34%	33%		30%	27%	
	% VHD	15%	13%		17%	14%		15%	13%	
Encounter severity	% unplanned	76%	78%	0.32	74%	80%	0.01	75%	77%	0.11
	Length of stay average	5.40	4.72	0.04	5.10	5.73	0.18	4.81	5.04	0.19
	% with ICU stay	22%	17%	0.04	21%	27%	0.03	19%	29%	<0.01
	% home health	38%	32%	0.03	39%	35%	0.23	33%	35%	0.19
Within-system cardio visits	Average # of within-system cardio visits in last year	5.19	4.40	<0.01	4.87	4.81	0.83	4.80	4.49	0.03

Encounters were assigned to time periods based on discharge date. Statistics are provided only for encounters meeting our filtering criteria. 2020 vs 2019 comparisons were performed using *t*-tests, with the exception of the encounter type and primary diagnosis comparisons which used chi-square tests. Within-system cardio visits refer to visits with a cardiovascular or cardiac surgery physician or advanced practice provider in the Mass General Brigham health system.

ACS = acute coronary syndrome; AD = arrhythmia disorders; ED = emergency department; HF = heart failure; ICU = intensive care unit; VHD = valvular heart disease.

### Unadjusted 14-day follow-up rates

**Figure 1:**
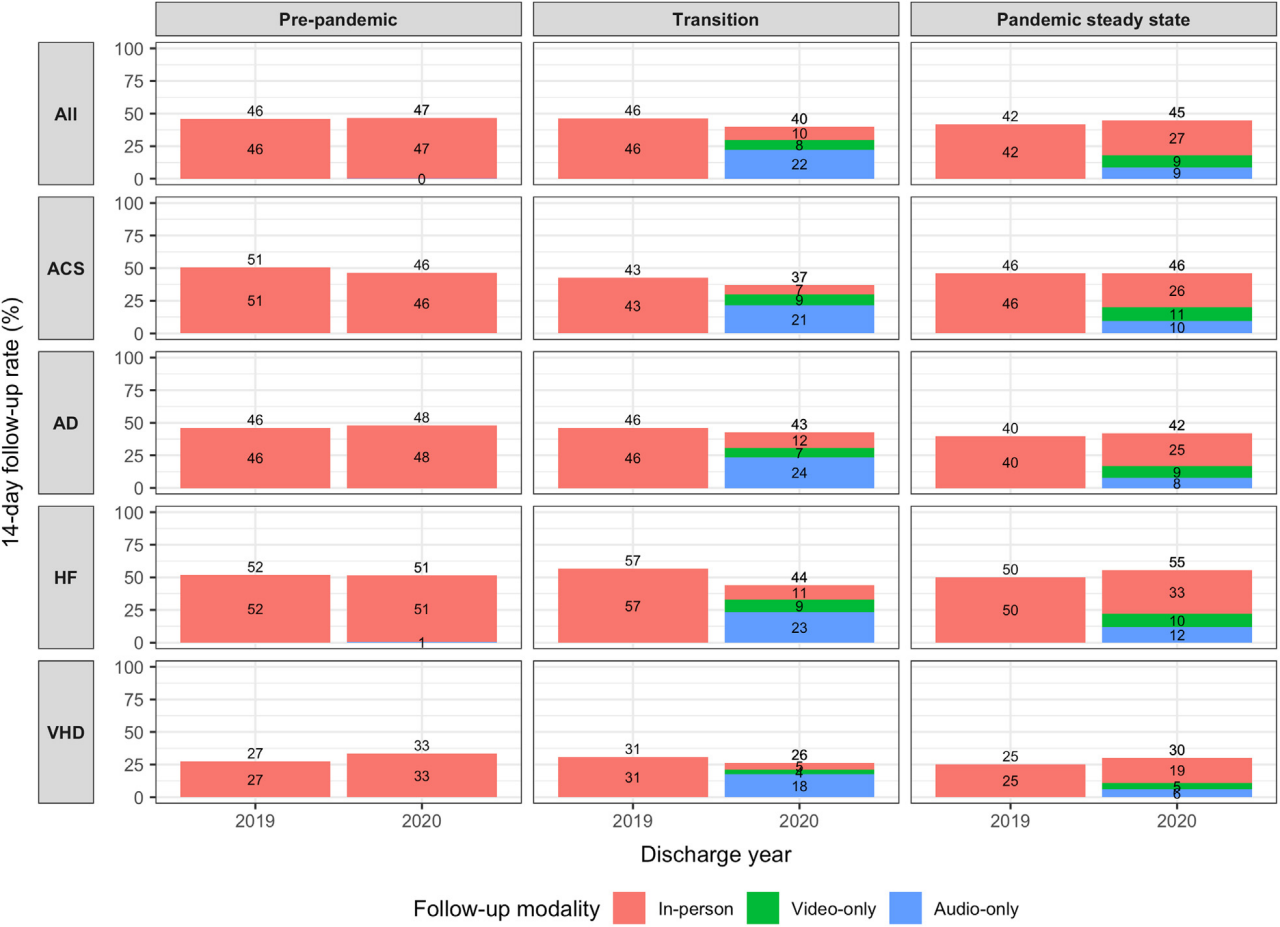
**Unadjusted 14-Day Follow-up Rates During the Prepandemic, Transition, and Pandemic Steady-State Time Periods** Follow-up is defined as a completed outpatient visit with a cardiovascular, cardiac surgery, or general internal medicine physician or advanced practice provider within 14 days of discharge. Follow-up modality denotes the most intensive visit type used within the 14 days following discharge (most intensive is in-person, while audio-only is least intensive). ACS = acute coronary syndrome; AD = arrhythmia disorders; HF = heart failure; VHD = valvular heart disease.

Figure 1 provides unadjusted 14-day follow-up rates for each 2020 period and the corresponding 2019 period. Follow-ups were categorized by modality—either in-person, video, or audio-only—with the 3 modality-specific follow-up rates summing to the aggregate rate. Rates are shown for the pooled set of encounters across conditions and for each condition separately.

During the 2020 prepandemic period, 46.7% of encounters had follow-up within 14 days of discharge, similar to the 46.0% follow-up rate in 2019 (difference: +0.8 percentage points [pp], 95% confidence interval [CI]: −5.3 to +6.8 pp, *P* = 0.80). In the 2020 and 2019 prepandemic periods, encounters were conducted almost entirely in-person.

During the 2020 transition period, 39.9% of encounters had a follow-up visit within 14 days of discharge compared to 46.5% in 2019 (difference: −6.5 pp, 95% CI: −12.4 to −0.7 pp, *P* = 0.03). In 2020, 29.9% of encounters had a 14-day follow-up using telemedicine (representing 75% of all follow-ups): 7.6% of encounters had video follow-ups (19% of follow-ups), and 22.3% of encounters had audio-only follow-ups (56% of follow-ups). In 2019, all follow-ups were conducted in-person.

In the 2020 pandemic steady-state period, 44.9% of encounters had a follow-up visit within 14 days of discharge compared to 41.7% in 2019 (difference: +3.2 pp, 95% CI: 0.0 to +6.3 pp, *P* = 0.05). In 2020, 18.0% of encounters had follow-ups using telemedicine (representing 40.0% of all follow-ups): 9.2% of encounters had video follow-ups (20% of follow-ups), and 8.9% of encounters had audio-only follow-ups (20% of follow-ups). In 2019, all follow-ups were conducted in-person. Examining the 4 conditions separately, we saw the largest increases for HF (50.1%-55.5%; difference: +5.4 pp, 95% CI: −0.6 to +11.3 pp, *P* = 0.08), moderate increases for VHD (25.3%-30.3%; difference: +4.9 pp, 95% CI: −2.7 to +12.5 pp, *P* = 0.20) and AD (39.8%-42.1%; difference: +2.3 pp, 95% CI: −2.7 to +7.3 pp, *P* = 0.37), while ACS showed little to no change (45.9%-45.9%; difference: 0.0 pp, 95% CI: −7.4 to +7.5 pp, *P* = 0.99). The proportion of follow-ups conducted via telemedicine were comparable across the 4 conditions.

### Adjusted 14-day follow-up rates

**Figure 2:**
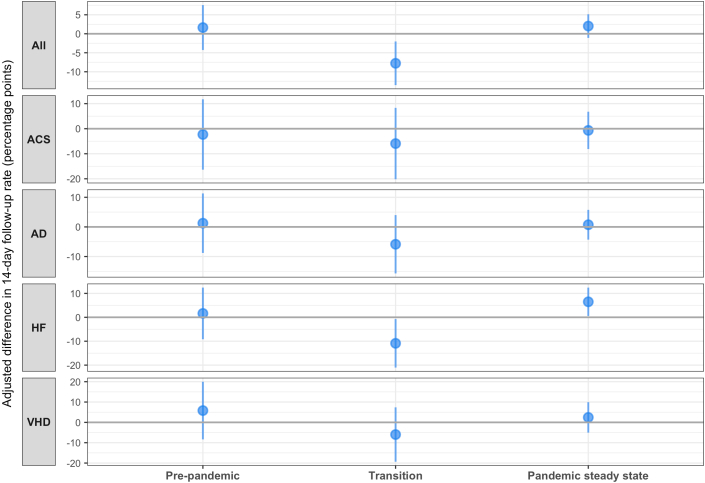
**Adjusted Difference in 14-Day Follow-up Rates, 2020 vs 2019** Point estimates represent differences in 14-day follow-up rate in 2020 vs 2019 in each of our 3 time periods. Estimates are adjusted for encounter and patient characteristics. Error bars reflect 95% confidence intervals with heteroskedasticity robust standard errors. ACS = acute coronary syndrome; AD = arrhythmia disorders; HF = heart failure; VHD = valvular heart disease.

After adjusting for encounter and patient characteristics, the increase in follow-up rate during the 2020 pandemic steady state across the pooled set of encounters was attenuated somewhat (adjusted difference: +2.0 pp, 95% CI: −1.1 to +5.1 pp, *P* = 0.20) (Figure 2). However, we continued to observe large increases in follow-up rate for HF encounters during the pandemic steady state (adjusted difference: +6.5 pp, 95% CI: +0.5 to +12.4 pp, *P* = 0.03). Results were qualitatively similar when examining adjusted differences by month (Supplemental Figure 3), with the majority of months after the transition period (June onwards) showing higher HF follow-up rates in 2020 relative to 2019. Results were also similar using 7-day, 21-day, and 28-day thresholds for timely follow-up (Supplemental Figure 4).

### Time to first follow-up

**Figure 3:**
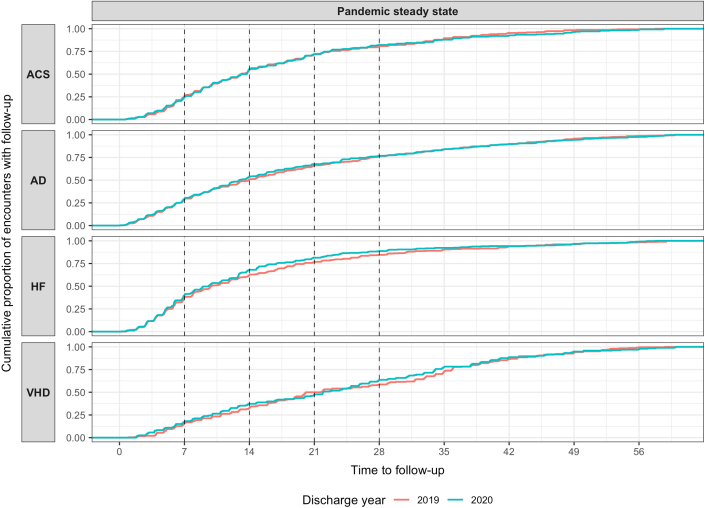
**Time to First Follow-up Cumulative Distribution Curves Stratified by Condition and Year** Only includes encounters from the pandemic steady state that had at least 1 follow-up visit within 60 days of discharge. Time to first follow-up is the number of days from discharge to the patients’ first follow-up. Follow-up defined as a completed outpatient visit with cardiovascular, cardiac surgery, or general internal medicine physician or advanced practice provider following discharge. ACS = acute coronary syndrome; AD = arrhythmia disorders; HF = heart failure; VHD = valvular heart disease.

Among pandemic steady-state ACS, AD, and VHD encounters with at least 1 follow-up visit within 60 days of discharge, time to follow-up distributions for 2020 and 2019 roughly matched each other; for HF, however, we observed excess density in the 14- to 28-day range in 2020 relative to 2019 (Figure 3, Supplemental Figure 5). In 2020, 67.6% of pandemic steady-state HF encounters with a follow-up within 60 days of discharge had their first follow-up in the first 14 days vs 62.7% in 2019 (*P* = 0.06), 81.4% had their follow-up within the first 21 days vs 76.7% in 2019 (*P* = 0.04), and 88.4% had their follow-up within 28 days relative to 84.5% in 2019 (*P* = 0.05) (Supplemental Table 3). By contrast, we observed only a 2.2-pp increase in the proportion of HF encounters that had a follow-up within 60 days in 2020 relative to that in 2019 (95% CI: −2.5 to 6.8 pp, *P* = 0.37). After adjusting for encounter and patient characteristics, we found that follow-ups after HF encounters from the 2020 pandemic steady state were on average 1.75 days earlier than those from the matched period in 2019 (95% CI: 0.02-3.48 days earlier, *P* = 0.05) (Supplemental Figure 6).

### 30-day all-cause unplanned readmission rates

**Figure 4:**
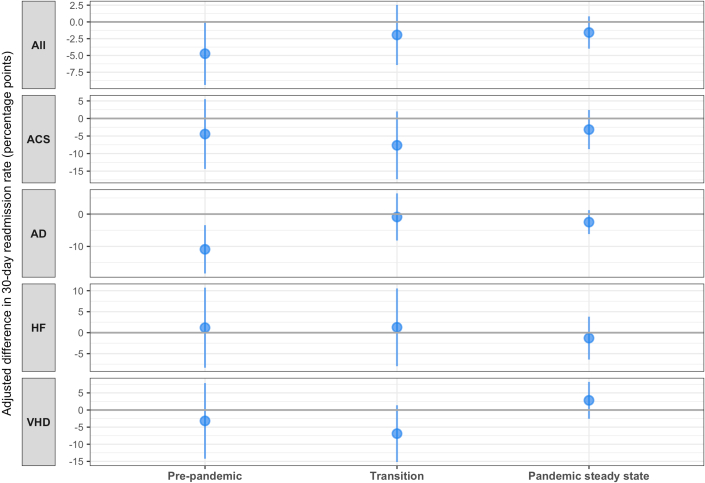
**Adjusted Difference in 30-Day All-Cause Unplanned Readmission Rate, 2020 vs 2019** Thirty-day unplanned readmissions include any emergency department, observation, or inpatient stays within 30 days of the index encounter discharge date. Readmissions are not restricted to those with cardiology primary diagnosis codes. Point estimates represent differences in readmission rates in each of our 3 time periods in 2020 vs 2019. Estimates are adjusted for encounter and patient characteristics. Error bars reflect 95% confidence intervals with heteroskedasticity robust standard errors. ACS = acute coronary syndrome; AD = arrhythmia disorders; HF = heart failure; VHD = valvular heart disease.

During the pandemic steady-state period, readmission rates remained relatively consistent with 2019 levels (Figure 4, Supplemental Figure 7). Overall readmission rates were down slightly from 2019, falling from 18.3% to 16.9% (adjusted difference −1.6 pp; 95% CI: −4.0 to +0.8 pp, *P* = 0.20). Slight decreases were seen for all conditions except VHD. Month-by-month comparisons showed qualitatively similar results through the second half of 2020 (Supplemental Figure 8).

## Discussion

Increased telemedicine use during the COVID-19 pandemic was associated with improvement in the timely follow-up rate after cardiovascular hospitalizations, primarily for HF encounters. Comparison of days to first follow-up suggests that the mechanism for this improvement was patients receiving earlier follow-ups, rather than a total increase in the number of follow-ups. In other words, there was little change in the likelihood of patients receiving follow-ups, but those who did receive them saw clinicians earlier.

Across the 4 acute cardiovascular conditions examined, HF encounters experienced the largest improvements in early follow-up rate. We note a few reasons why this may be the case. First, HF patients are most likely to benefit from timely follow-up given their poor prognosis after discharge and high risk of early readmission. Among Medicare beneficiaries, 1-year mortality following a HF hospitalization is estimated to be around 30%.^27^ Additionally, 30-day readmission rates for HF hospitalizations are among the highest for any condition, ranging from 20% to 25%.^28^ Given the high risk for this population, both U.S. and European guidelines recommend follow-up within 2 weeks of discharge.^25^^,^^26^ Second, readmissions following HF encounters can have important financial impacts on hospitals. In 2012, Centers for Medicare & Medicaid Services established the Hospital Readmissions Reduction Program (HRRP), which applied financial penalties to hospitals with excess 30-day readmissions among Medicare beneficiaries. In 2019, HRRP penalties totaled $563 million.^29^ Hospitals may therefore be financially incentivized to prioritize HF patients for timely follow-up over other cardiovascular patient cohorts given constraints on clinician time.

In 2019, prior to the pandemic, follow-up patterns for HF encounters were meaningfully different from those for our other 3 conditions (Supplemental Table 4). At baseline, HF encounters had earlier first follow-ups and higher 14-day and 28-day follow-up rates. HF encounters also had a higher likelihood of having their first postdischarge follow-up appointment booked during the hospitalization. These particular follow-ups were conducted on average within 9.2 days from the discharge date. Additionally, follow-ups for HF encounters were less likely to be with a cardiologist and more likely to be with an advanced practice provider. These findings suggest that speed to follow-up was prioritized over the type of clinician the patient followed-up with.

Our results suggest that, when early follow-up is prioritized, telemedicine can be a useful tool for improving the provision of outpatient follow-ups after acute cardiovascular hospital encounters. Improvements in patient access to timely follow-up can have positive impacts on patients' quality of life and adherence to care plans.^15^^,^^19^ Additionally, replacing in-person care with telemedicine visits can have meaningful productivity, cost, and convenience benefits for patients, clinicians, and health systems.^3^, ^4^, ^5^^,^^7^

Follow-up care quality is another important dimension that may be impacted by increased telemedicine use. While noise in our readmission rate measures and sample size constraints limited our ability to detect smaller changes to readmission rates, we were able to rule out dramatic increases in readmissions. Our findings, consistent with other recent literature,^7^^,^^30^ suggests that the shift to telemedicine during the pandemic did not have a substantial effect on the quality of follow-up care as measured by unplanned readmissions. During the pandemic steady state, despite 40% of follow-ups shifting from traditional in-person visits to telemedicine visits, readmission rates overall were comparable to those in the matched period in 2019. If there were negative quality impacts of conducting follow-ups via telemedicine, they were either not captured in readmission rates, such as other effects on patient engagement or quality of life, or were too small to detect in our study.

Finally, while our results showed improvements in the follow-up rate coinciding with increased telemedicine availability and no dramatic changes in care quality as measured by unplanned readmissions, they do not imply that telemedicine would be an appropriate substitute for in-person care for all types of follow-up. This may be true not only for clinical reasons but also because some patients face barriers to accessing telemedicine due to a lack of technology or bandwidth.^11^, ^12^, ^13^ During the pandemic steady-state period, 60% of outpatient follow-ups were still conducted in-person. It is possible that triaging by clinicians influenced the assignment of patients to appropriate modalities of care. Further research may help identify the types of patients or encounters that benefit most from telemedicine.

### Study Limitations

Our study should be interpreted with several limitations in mind. First, because our data were drawn from a single health system, we were unable to observe follow-ups occurring outside the system. This concern is partially mitigated by restricting our study population to patients with established cardiovascular care within the MGB health system in the 2 years prior to the index encounter, as these patients are less likely to receive care from clinicians outside MGB. Additionally, as long as the rate of out-of-system follow-ups was relatively consistent in 2019 and 2020, we would not expect bias in our estimates.

Second, our reliance on data from a single health system may limit the generalizability of our findings. Although MGB includes a wide range of care entities including large urban academic hospitals and smaller rural community hospitals, its patient population is drawn predominantly from New England and upstate New York and includes a lower proportion of non-White patients and a higher proportion of patients from high-income zip codes than the U.S. population as a whole. There may be digital access barriers for minority and low-income populations which cannot be captured in our study.

Third, our analysis of all-cause readmission rates provides only a preliminary perspective on follow-up care quality. As we noted, inherent noise in readmission measures along with our limited sample size constrained the precision of our estimates and warrants further investigation with a larger data set. Additionally, all-cause readmission does not capture other important aspects of care quality such as patient satisfaction. However, all-cause readmission rates are still an important measure for patient outcomes and hospital financial performance through HRRP.

Fourth, our study population excluded encounters where the patient was discharged to post–acute care facilities or hospice. These encounters may have experienced distinct impacts from the shift to telemedicine.

Finally, because the increase in telemedicine use coincided with the COVID-19 pandemic, it is difficult to distinguish the effects of telemedicine use from those of the pandemic broadly. Our focus on the steady-state period—when MGB hospital encounter and outpatient volumes had largely returned to prepandemic levels—and adjustments to our estimates based on encounter and patient characteristics should mitigate this concern somewhat.

## Conclusions

Our results from a large integrated health system supports the use of telemedicine as a tool for improving patient access to timely follow-ups after acute cardiovascular hospital encounters (Central Illustration). We saw this most dramatically for HF patients, where timely follow-ups are most critical among the 4 conditions we studied. Future research providing more granular identification of which types of encounters are best suited for telemedicine follow-ups will be valuable in enhancing telemedicine’s beneficial impact on care delivery.

**Central Illustration:**
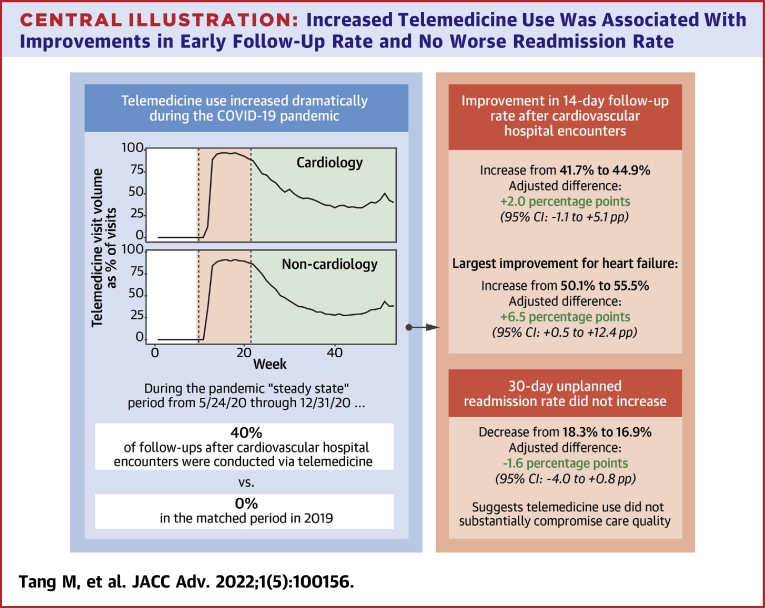
**Increased Telemedicine Use Was Associated With Improvements in Early Follow-Up Rate and No Worse Readmission Rate** Acute cardiovascular hospital encounters include encounters for acute coronary syndrome, arrhythmia disorders, heart failure, and valvular heart disease resulting in hospitalizations, emergency department (ED) visits, or observation stays. The **graph in the top left** shows 2020 weekly telemedicine visit volume as a percentage of total outpatient visit volume for cardiovascular or cardiac surgery physicians and advanced practice providers (APPs) **(top)** and general internal medicine physicians and APPs **(bottom)**. The **red** highlighted weeks correspond to the pandemic “transition” period (March 1, 2020-May 23, 2020), while the **green** highlighted weeks correspond to the pandemic “steady state” period (May 24, 2020-December 31, 2020). Telemedicine visits include both video and audio-only visits. Fourteen-day follow-up is defined as a completed outpatient visit with a cardiovascular, cardiac surgery, or general internal medicine physician or APP within 14 days of discharge. Thirty-day unplanned readmissions include any ED, observation, or inpatient stays within 30 days of the index encounter discharge date. Readmissions are not restricted to those with cardiology primary diagnosis codes. Percentage point (pp) changes shown on the right reflect adjusted differences between encounters from the 2020 pandemic steady-state period and the week-matched period in 2019. Estimates are adjusted for both encounter and patient characteristics, and 95% confidence intervals are provided below the estimates.

## Funding support and author disclosures

Dr Bhatt has received consulting fees from Sanofi Pasteur and Verve Therapeutics; served on the medical advisory board of Cohere Health; and is supported by the 10.13039/100000050National Heart, Lung, and Blood Institute T32 postdoctoral training grant T32HL094301. Dr Varshney has received support from the 10.13039/100000050National Heart, Lung, and Blood Institute T32 postdoctoral training grant T32HL007604 and the Daniel Pierce Family Fellowship in Advanced Heart Disease; served on the Advisory Board for Broadview Ventures; and received consulting fees from Buoy Health, Inc. Dr Vaduganathan has received research grant support or served on the advisory boards for American Regent, Amgen, AstraZeneca, Bayer AG, Baxter Healthcare, Boehringer Ingelheim, Cytokinetics, Lexicon Pharmaceuticals, Novartis, Pharmacosmos, Relypsa, Roche Diagnostics, and Sanofi; has speaker engagements with Novartis and Roche Diagnostics; and participates on clinical trial committees for studies sponsored by Galmed, Novartis, Bayer AG, Occlutech, and Impulse Dynamics. Dr Huckman’s research is funded by the 10.13039/100007300Harvard Business School’s Division of Research and Faculty Development; he serves as an advisory board member for RubiconMD, Arena, and Carrum Health; he also serves as an uncompensated trustee of the Brigham and Women’s Physician Organization and Brigham Health; and he receives compensation for teaching in a Harvard Business School executive education program for Brigham Health. All other authors have reported that they have no relationships relevant to the contents of this paper to disclose.

PERSPECTIVES **COMPETENCY IN MEDICAL KNOWLEDGE:** Our results point to telemedicine as a promising tool for expanding patient access to timely follow-ups after acute cardiovascular hospitalizations.

**TRANSLATIONAL OUTLOOK:** Future research providing more granular identification of which types of encounters are best suited for telemedicine follow-ups will be valuable to enhancing telemedicine’s beneficial impact on care delivery.
