# Supplementary material for: Investigating the Association Between Telemedicine Use and Timely Follow-Up Care After Acute Cardiovascular Hospital Encounters
Source: JACC Adv. 2022 Dec 30;1(5):100156. doi: 10.1016/j.jacadv.2022.100156 (PMC9802537; doi:10.1016/j.jacadv.2022.100156)
Supplement: Supplemental Figures S1–S8 and Tables S1–S4 [file mmc1.docx]

**Supplemental Table 1.** ICD-10 primary diagnosis codes identifying cardiovascular conditions of interest

| **Cardiovascular condition** | **ICD-10 Code** | **Description** |
| --- | --- | --- |
| Acute coronary syndrome | I20.0 | Unstable angina |
|  | I20.1 | Angina pectoris with documented spasm |
|  | I20.8 | Other forms of angina pectoris |
|  | I20.9 | Angina pectoris, unspecified |
|  | I21.01 | ST elevation (STEMI) myocardial infarction involving left main coronary artery |
|  | I21.02 | ST elevation (STEMI) myocardial infarction involving left anterior descending coronary artery |
|  | I21.09 | ST elevation (STEMI) myocardial infarction involving other coronary artery of anterior wall |
|  | I21.11 | ST elevation (STEMI) myocardial infarction involving right coronary artery |
|  | I21.19 | ST elevation (STEMI) myocardial infarction involving other coronary artery of inferior wall |
|  | I21.21 | ST elevation (STEMI) myocardial infarction involving left circumflex coronary artery |
|  | I21.29 | ST elevation (STEMI) myocardial infarction involving other sites |
|  | I21.3 | ST elevation (STEMI) myocardial infarction of unspecified site |
|  | I21.4 | Non-ST elevation (NSTEMI) myocardial infarction |
|  | I21.9 | Acute myocardial infarction, unspecified |
|  | I22.2 | Subsequent non-ST elevation (NSTEMI) myocardial infarction |
|  | I23.7 | Postinfarction angina |
|  | I24.0 | Acute coronary thrombosis not resulting in myocardial infarction |
|  | I24.8 | Other forms of acute ischemic heart disease |
|  | I24.9 | Acute ischemic heart disease, unspecified |
|  | I25.10 | Atherosclerotic heart disease of native coronary artery without angina pectoris |
|  | I25.10, I25.82 | Atherosclerotic heart disease of native coronary artery without angina pectoris, Chronic total occlusion of coronary artery |
|  | I25.10, I25.83 | Atherosclerotic heart disease of native coronary artery without angina pectoris, Coronary atherosclerosis due to lipid rich plaque |
|  | I25.10, I25.84 | Atherosclerotic heart disease of native coronary artery without angina pectoris, Coronary atherosclerosis due to calcified coronary lesion |
|  | I25.10, Z98.61 | Atherosclerotic heart disease of native coronary artery without angina pectoris, Coronary angioplasty status |
|  | I25.110 | Atherosclerotic heart disease of native coronary artery with unstable angina pectoris |
|  | I25.111 | Atherosclerotic heart disease of native coronary artery with angina pectoris with documented spasm |
|  | I25.118 | Atherosclerotic heart disease of native coronary artery with other forms of angina pectoris |
|  | I25.119 | Atherosclerotic heart disease of native coronary artery with unspecified angina pectoris |
|  | I25.2 | Old myocardial infarction |
|  | I25.3 | Aneurysm of heart |
|  | I25.41 | Coronary artery aneurysm |
|  | I25.42 | Coronary artery dissection |
|  | I25.700 | Atherosclerosis of coronary artery bypass graft(s), unspecified, with unstable angina pectoris |
|  | I25.708 | Atherosclerosis of coronary artery bypass graft(s), unspecified, with other forms of angina pectoris |
|  | I25.709 | Atherosclerosis of coronary artery bypass graft(s), unspecified, with unspecified angina pectoris |
|  | I25.720 | Atherosclerosis of autologous artery coronary artery bypass graft(s) with unstable angina pectoris |
|  | I25.729 | Atherosclerosis of autologous artery coronary artery bypass graft(s) with unspecified angina pectoris |
|  | I25.750 | Atherosclerosis of native coronary artery of transplanted heart with unstable angina |
|  | I25.758 | Atherosclerosis of native coronary artery of transplanted heart with other forms of angina pectoris |
|  | I25.810 | Atherosclerosis of coronary artery bypass graft(s) without angina pectoris |
|  | I25.82 | Chronic total occlusion of coronary artery |
|  | I25.84 | Coronary atherosclerosis due to calcified coronary lesion |
|  | I25.9 | Chronic ischemic heart disease, unspecified |
|  | Z95.1 | Presence of aortocoronary bypass graft |
|  | Z95.5 | Presence of coronary angioplasty implant and graft |
|  | Z98.61 | Coronary angioplasty status |
| Arrhythmia disorders | I44.0 | Atrioventricular block, first degree |
|  | I44.1 | Atrioventricular block, second degree |
|  | I44.2 | Atrioventricular block, complete |
|  | I44.30 | Unspecified atrioventricular block |
|  | I44.39 | Other atrioventricular block |
|  | I45.4 | Nonspecific intraventricular block |
|  | I45.5 | Other specified heart block |
|  | I45.6 | Pre-excitation syndrome |
|  | I45.81 | Long QT syndrome |
|  | I45.89 | Other specified conduction disorders |
|  | I45.9 | Conduction disorder, unspecified |
|  | I47.1 | Supraventricular tachycardia |
|  | I47.2 | Ventricular tachycardia |
|  | I47.9 | Paroxysmal tachycardia, unspecified |
|  | I48.0 | Paroxysmal atrial fibrillation |
|  | I48.11 | Longstanding persistent atrial fibrillation |
|  | I48.19 | Other persistent atrial fibrillation |
|  | I48.20 | Chronic atrial fibrillation, unspecified |
|  | I48.21 | Permanent atrial fibrillation |
|  | I48.3 | Typical atrial flutter |
|  | I48.4 | Atypical atrial flutter |
|  | I48.91 | Unspecified atrial fibrillation |
|  | I48.92 | Unspecified atrial flutter |
|  | I49.3 | Ventricular premature depolarization |
|  | I49.5 | Sick sinus syndrome |
|  | I49.8 | Other specified cardiac arrhythmias |
|  | I49.9 | Cardiac arrhythmia, unspecified |
|  | T82.110 | Breakdown (mechanical) of cardiac electrode |
|  | T82.111 | Breakdown (mechanical) of cardiac pulse generator (battery) |
|  | T82.118 | Breakdown (mechanical) of other cardiac electronic device |
|  | T82.119 | Breakdown (mechanical) of unspecified cardiac electronic device |
|  | T82.120 | Displacement of cardiac electrode |
|  | T82.121 | Displacement of cardiac pulse generator (battery) |
|  | T82.128 | Displacement of other cardiac electronic device |
|  | T82.129 | Displacement of unspecified cardiac electronic device |
|  | T82.190 | Other mechanical complication of cardiac electrode |
|  | T82.191 | Other mechanical complication of cardiac pulse generator (battery) |
|  | T82.198 | Other mechanical complication of other cardiac electronic device |
|  | T82.199 | Other mechanical complication of unspecified cardiac device |
|  | Z45.010 | Encounter for checking and testing of cardiac pacemaker pulse generator [battery] |
|  | Z45.018 | Encounter for adjustment and management of other part of cardiac pacemaker |
|  | Z45.02 | Encounter for adjustment and management of automatic implantable cardiac defibrillator |
|  | Z45.09 | Encounter for adjustment and management of other cardiac device |
|  | Z95.0 | Presence of cardiac pacemaker |
|  | Z95.810 | Presence of automatic (implantable) cardiac defibrillator |
| Heart failure | I11.0 | Hypertensive heart disease with heart failure |
|  | I25.5 | Ischemic cardiomyopathy |
|  | I25.5, I42.0 | Ischemic cardiomyopathy, Dilated cardiomyopathy |
|  | I50.1 | Left ventricular failure, unspecified |
|  | I50.20 | Unspecified systolic (congestive) heart failure |
|  | I50.21 | Acute systolic (congestive) heart failure |
|  | I50.22 | Chronic systolic (congestive) heart failure |
|  | I50.23 | Acute on chronic systolic (congestive) heart failure |
|  | I50.30 | Unspecified diastolic (congestive) heart failure |
|  | I50.31 | Acute diastolic (congestive) heart failure |
|  | I50.32 | Chronic diastolic (congestive) heart failure |
|  | I50.33 | Acute on chronic diastolic (congestive) heart failure |
|  | I50.40 | Unspecified combined systolic (congestive) and diastolic (congestive) heart failure |
|  | I50.41 | Acute combined systolic (congestive) and diastolic (congestive) heart failure |
|  | I50.42 | Chronic combined systolic (congestive) and diastolic (congestive) heart failure |
|  | I50.43 | Acute on chronic combined systolic (congestive) and diastolic (congestive) heart failure |
|  | I50.9 | Heart failure, unspecified |
|  | Z95.811 | Presence of heart assist device |
| Valvular heart disease | I05.0 | Rheumatic mitral stenosis |
|  | I05.1 | Rheumatic mitral insufficiency |
|  | I05.2 | Rheumatic mitral stenosis with insufficiency |
|  | I05.8 | Other rheumatic mitral valve diseases |
|  | I05.9 | Rheumatic mitral valve disease, unspecified |
|  | I06.0 | Rheumatic aortic stenosis |
|  | I06.1 | Rheumatic aortic insufficiency |
|  | I06.2 | Rheumatic aortic stenosis with insufficiency |
|  | I06.8 | Other rheumatic aortic valve diseases |
|  | I06.9 | Rheumatic aortic valve disease, unspecified |
|  | I07.0 | Rheumatic tricuspid stenosis |
|  | I07.1 | Rheumatic tricuspid insufficiency |
|  | I07.2 | Rheumatic tricuspid stenosis and insufficiency |
|  | I07.8 | Other rheumatic tricuspid valve diseases |
|  | I07.9 | Rheumatic tricuspid valve disease, unspecified |
|  | I08.0 | Rheumatic disorders of both mitral and aortic valves |
|  | I08.1 | Rheumatic disorders of both mitral and tricuspid valves |
|  | I08.2 | Rheumatic disorders of both aortic and tricuspid valves |
|  | I08.3 | Combined rheumatic disorders of mitral, aortic and tricuspid valves |
|  | I08.8 | Other rheumatic multiple valve diseases |
|  | I08.9 | Rheumatic multiple valve disease, unspecified |
|  | I34.0 | Nonrheumatic mitral (valve) insufficiency |
|  | I34.1 | Nonrheumatic mitral (valve) prolapse |
|  | I34.2 | Nonrheumatic mitral (valve) stenosis |
|  | I34.8 | Other nonrheumatic mitral valve disorders |
|  | I34.9 | Nonrheumatic mitral valve disorder, unspecified |
|  | I35.0 | Nonrheumatic aortic (valve) stenosis |
|  | I35.1 | Nonrheumatic aortic (valve) insufficiency |
|  | I35.2 | Nonrheumatic aortic (valve) stenosis with insufficiency |
|  | I35.8 | Other nonrheumatic aortic valve disorders |
|  | I35.9 | Nonrheumatic aortic valve disorder, unspecified |
|  | I36.0 | Nonrheumatic tricuspid (valve) stenosis |
|  | I36.1 | Nonrheumatic tricuspid (valve) insufficiency |
|  | I36.2 | Nonrheumatic tricuspid (valve) stenosis with insufficiency |
|  | I36.8 | Other nonrheumatic tricuspid valve disorders |
|  | I36.9 | Nonrheumatic tricuspid valve disorder, unspecified |
|  | I37.0 | Nonrheumatic pulmonary valve stenosis |
|  | I37.1 | Nonrheumatic pulmonary valve insufficiency |
|  | I37.2 | Nonrheumatic pulmonary valve stenosis with insufficiency |
|  | I37.8 | Other nonrheumatic pulmonary valve disorders |
|  | I37.9 | Nonrheumatic pulmonary valve disorder, unspecified |
|  | T82.01 | Breakdown (mechanical) of heart valve prosthesis |
|  | T82.02 | Displacement of heart valve prosthesis |
|  | T82.03 | Leakage of heart valve prosthesis |
|  | T82.09 | Other mechanical complication of heart valve prosthesis |
|  | T82.221 | Breakdown (mechanical) of biological heart valve graft |
|  | T82.222 | Displacement of biological heart valve graft |
|  | T82.223 | Leakage of biological heart valve graft |
|  | T82.228 | Other mechanical complication of biological heart valve graft |
|  | T82.6 | Infection and inflammatory reaction due to cardiac valve prosthesis |
|  | Z95.2 | Presence of prosthetic heart valve |
|  | Z95.3 | Presence of xenogenic heart valve |
|  | Z95.4 | Presence of other heart-valve replacement |

**Supplemental Figure 1.** 2020 weekly telemedicine visit volume as a % of total outpatient visit volume


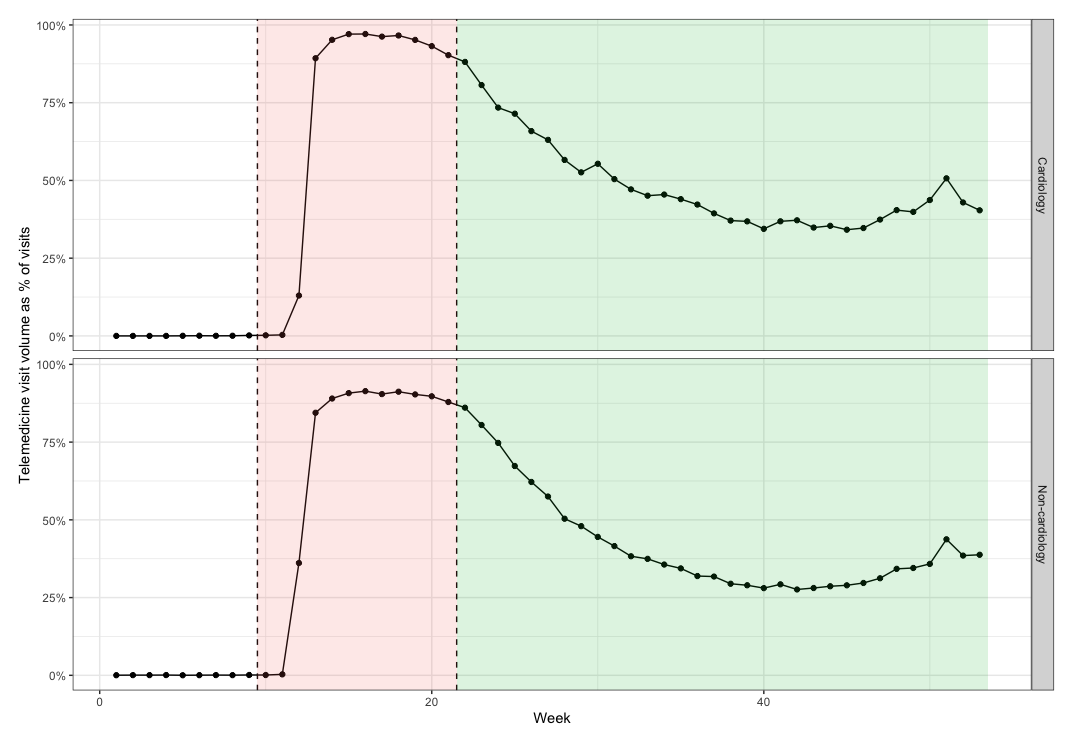


**Notes**: Telemedicine visits include both video and audio-only visits. Total outpatient visit volume is not limited to post-hospitalization follow-ups. The red highlighted weeks correspond to the transition period, while the green highlighted weeks correspond to the pandemic steady state period. The top half of the plot reflects telemedicine use by cardiology or cardiac surgery physicians and advanced practice providers (APPs). The bottom half of the plot reflects telemedicine use by general internal medicine physicians and APPs.

**Supplemental Figure 2.** 2020 weekly audio-only telemedicine visit volume as a % of total telemedicine visit volume


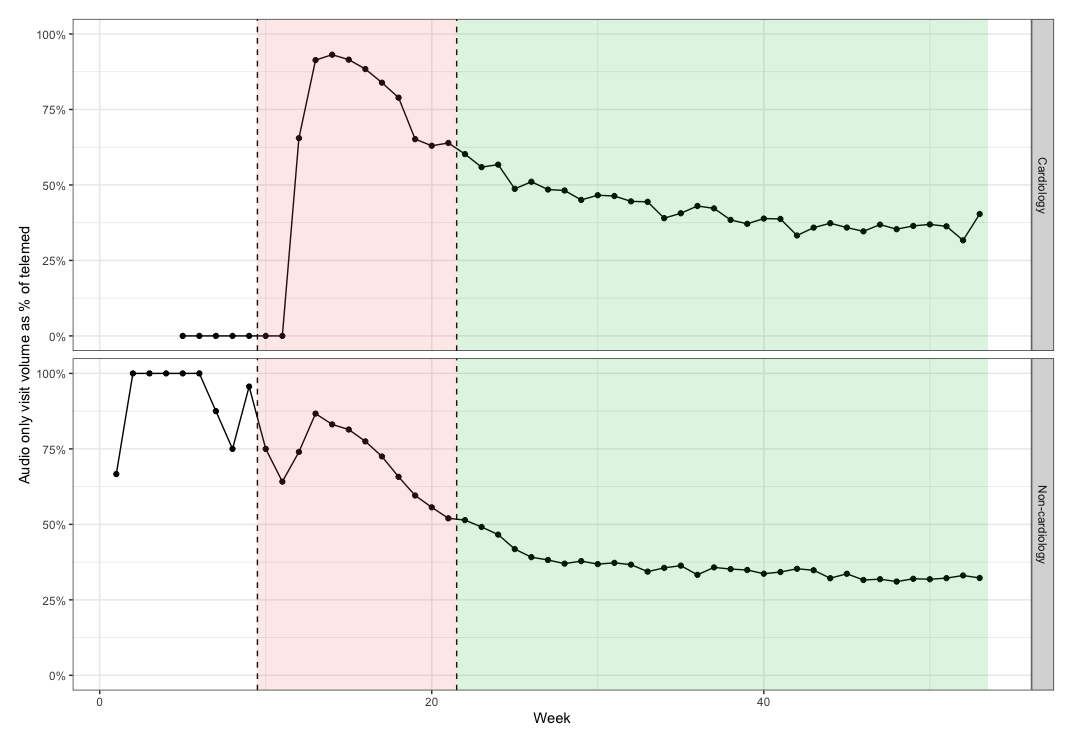


**Notes**: Total telemedicine visits include both video and audio-only visits. Total telemedicine visit volume is not limited to post-hospitalization follow-ups. The red highlighted weeks correspond to the transition period, while the green highlighted weeks correspond to the pandemic steady state period. The top half of the plot reflects telemedicine use by cardiology or cardiac surgery physicians and advanced practice providers (APPs). The bottom half of the plot reflects telemedicine use by general internal medicine physicians and APPs.

**Supplemental Table 2**. Encounter filtering criteria

| **Filters** | **Inpatient** | **ED** | **Observation** | **Total** |
| --- | --- | --- | --- | --- |
| **1. Encounter discharge date between 1/1/19 and 12/31/20** | 33,562 | 17,290 | 6,012 | **56,864** |
| **2. Primary ICD-10 diagnosis on our list of cardiovascular conditions** | 13,835 | 2,454 | 1,047 | **17,336** |
| **3. Include only index encounters  (exclude any hospital visits within 30 days of a prior hospital visit)** | 10,370 | 2,009 | 816 | **13,195** |
| **4. Patient had 1+ within-system cardiology outpatient visits within 24 months prior to the index encounter** | 5,949 | 892 | 420 | **7,261** |
| **5. Patient discharged to home** | **4,923** | **703** | **400** | **6,026** |

**Notes**: Data in each cell reflect the number of encounters passing the filter in the corresponding row as well as all filters on prior rows. The final row reflects the total number of encounters passing our filtering criteria.

**Supplemental Figure 3.** 2020 vs. 2019 adjusted difference in 14-day follow-up rates by month


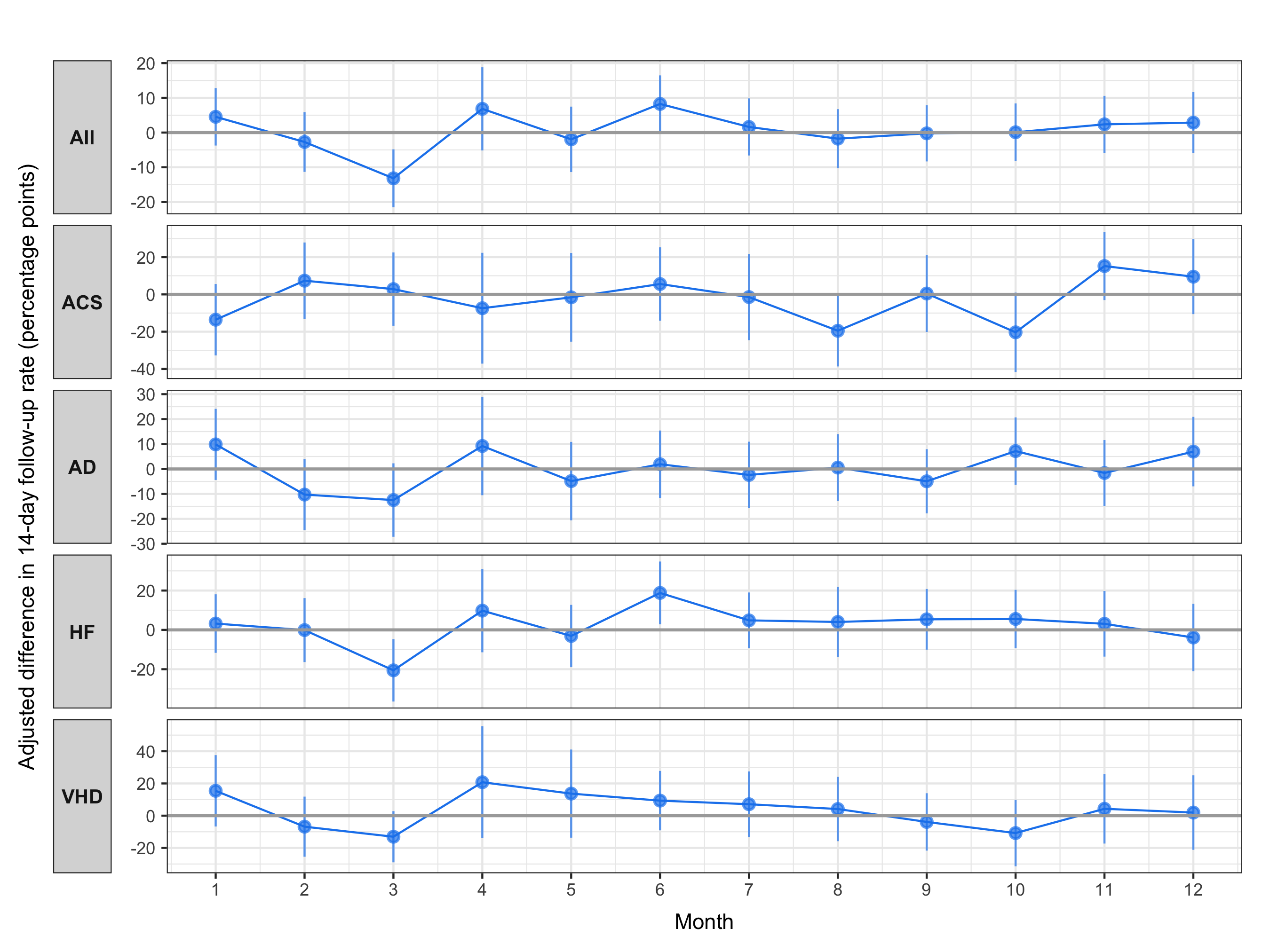


**Notes**: Point estimates represent differences in 14-day follow-up rate in 2020 vs. 2019 by month. Estimates are adjusted for encounter and patient characteristics. Error bars reflect 95% confidence intervals with heteroskedasticity robust standard errors. ACS = acute coronary syndrome; AD = arrhythmia disorders; HF = heart failure; VHD = valvular heart disease.

**Supplemental Figure 4.** 2020 vs. 2019 adjusted difference in follow-up rates – follow-up threshold comparison


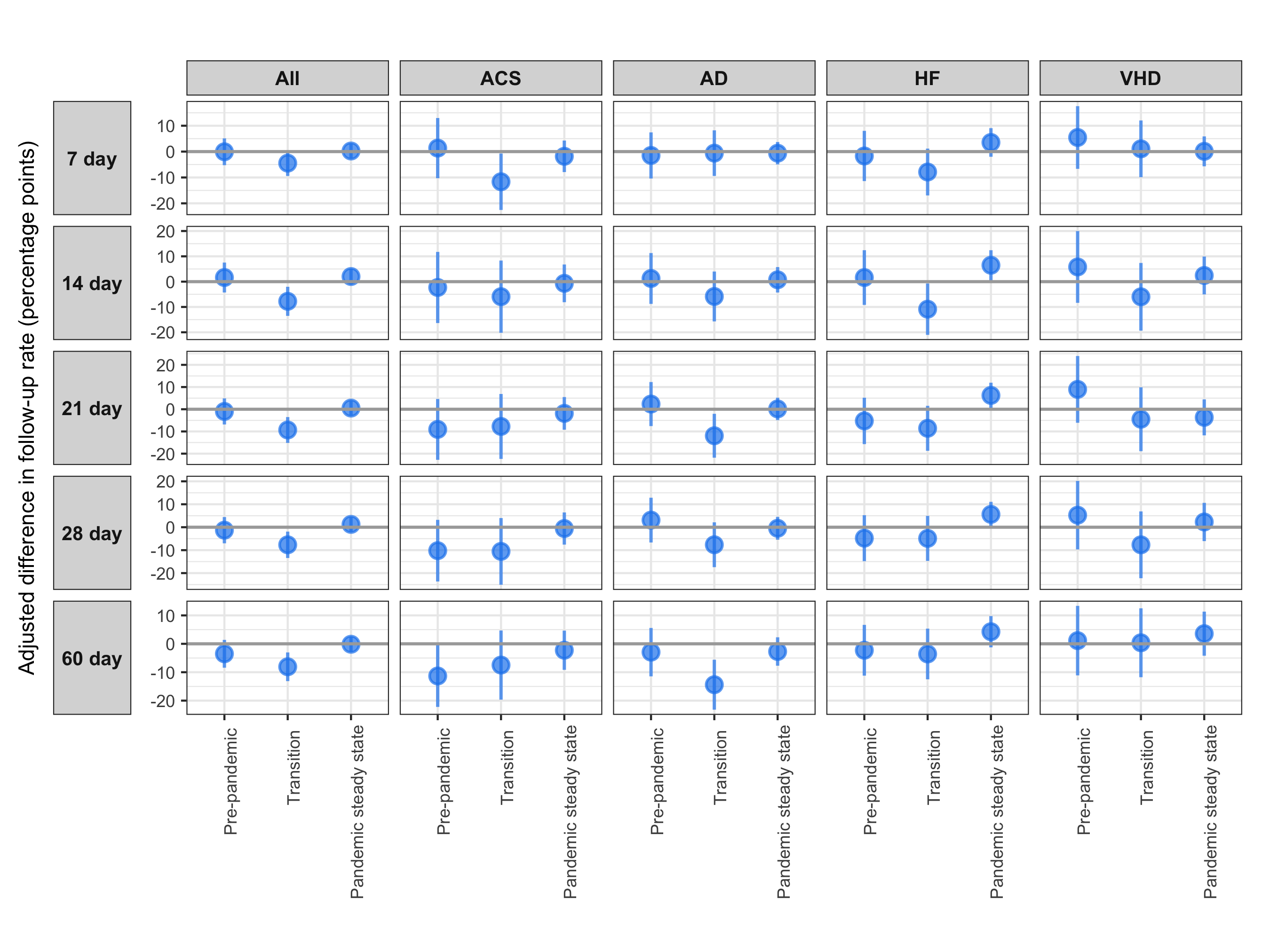


**Notes**: Point estimates represent differences in follow-up rate in 2020 vs. 2019 in each of our three time periods across various conditions and follow-up period combinations. Estimates are adjusted for encounter and patient characteristics. Error bars reflect 95% confidence intervals with heteroskedasticity robust standard errors. ACS = acute coronary syndrome; AD = arrhythmia disorders; HF = heart failure; VHD = valvular heart disease.

**Supplemental Figure 5.** Kernel density plots of time to first follow-up among encounters with ≥1 follow-up within 60 days


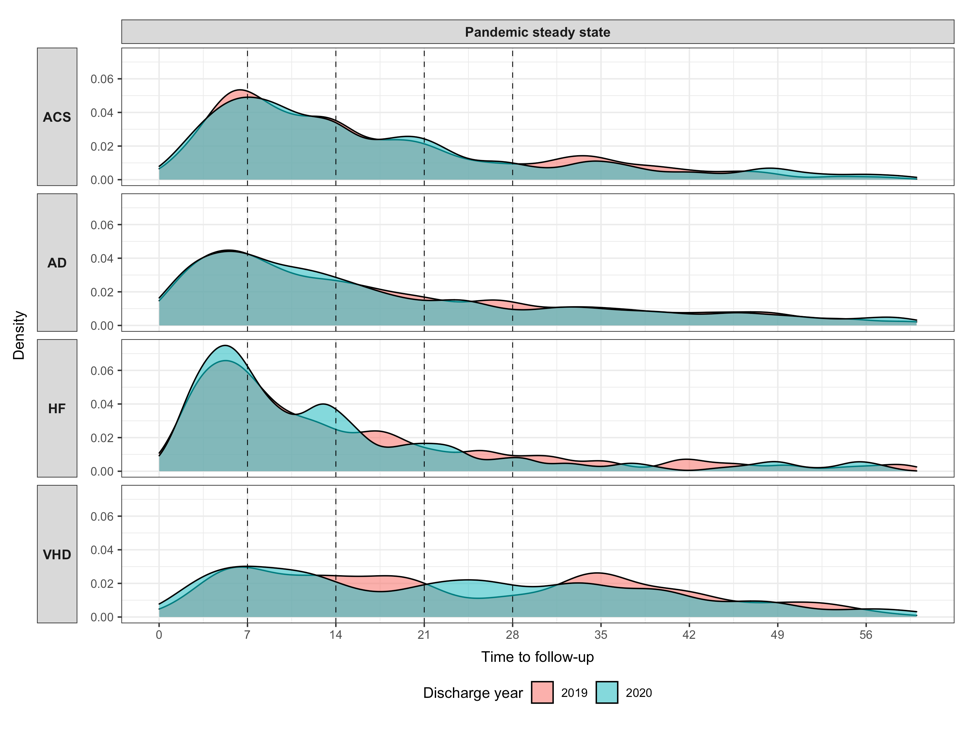


**Notes**: Data reflects encounters from the pandemic steady state that had at least one follow-up within 60 days of discharge. Time to follow-up is the number of days from discharge to the patients' first follow-up. Follow-up defined as a completed outpatient visit with a cardiovascular, cardiac surgery, or general internal medicine physician or advanced practice provider. Densities are calculated based on a gaussian kernel with bandwidth half that recommended by Silverman’s ‘rule of thumb’. ACS = acute coronary syndrome; AD = arrhythmia disorders; HF = heart failure; VHD = valvular heart disease.

**Supplemental Table 3.** Cumulative distribution comparison at 7, 14, 21, and 28 days since discharge

|  |  | **% of encounters with first follow-up by that point** | |  |  |
| --- | --- | --- | --- | --- | --- |
| **Condition** | **Days to first follow-up** | **2019** | **2020** | **Difference** | **P-value** |
| **ACS** | 7 days | 25.8% | 24.5% | -1.4% | 0.649 |
|  | 14 days | 54.7% | 55.8% | 1.1% | 0.392 |
|  | 21 days | 71.8% | 71.9% | 0.1% | 0.491 |
|  | 28 days | 80.2% | 82.1% | 1.9% | 0.279 |
|  | *60 day fu rate* | *83.9%* | *82.3%* | *-1.7%* | *0.562* |
| **AD** | 7 days | 29.3% | 29.1% | -0.1% | 0.518 |
|  | 14 days | 50.9% | 53.6% | 2.8% | 0.172 |
|  | 21 days | 66.2% | 67.7% | 1.5% | 0.292 |
|  | 28 days | 76.2% | 76.7% | 0.5% | 0.414 |
|  | *60 day fu rate* | *78.3%* | *78.5%* | *0.2%* | *0.916* |
| **HF** | 7 days | 37.3% | 39.9% | 2.6% | 0.212 |
|  | 14 days | 62.7% | 67.6% | 4.9% | 0.064 |
|  | 21 days | 76.7% | 81.4% | 4.7% | 0.044 |
|  | 28 days | 84.5% | 88.4% | 3.9% | 0.047 |
|  | *60 day fu rate* | *79.9%* | *82.1%* | *2.2%* | *0.365* |
| **VHD** | 7 days | 16.0% | 17.1% | 1.1% | 0.378 |
|  | 14 days | 32.4% | 37.3% | 4.9% | 0.141 |
|  | 21 days | 50.0% | 47.2% | -2.8% | 0.723 |
|  | 28 days | 58.6% | 63.2% | 4.6% | 0.164 |
|  | *60 day fu rate* | *78.2%* | *81.1%* | *2.9%* | *0.404* |

**Notes**: Data reflects encounters from the pandemic steady state that had at least one follow-up within 60 days of discharge. “% of encounters with first follow-up by that point” match the values shown in Figure 3. The columns titled “2019” and “2020” reflect the % of encounters within that particular year that had a follow-up within a given number of days from discharge. An additional row is included for each condition comparing the 60-day follow-up rate between 2019 and 2020 (i.e., the denominator for the prior rows). ACS = acute coronary syndrome; AD = arrhythmia disorders; HF = heart failure; VHD = valvular heart disease.

**Supplemental Figure 6.** 2020 vs. 2019 adjusted difference in time to first follow-up among encounters with ≥1 follow-up within 60 days


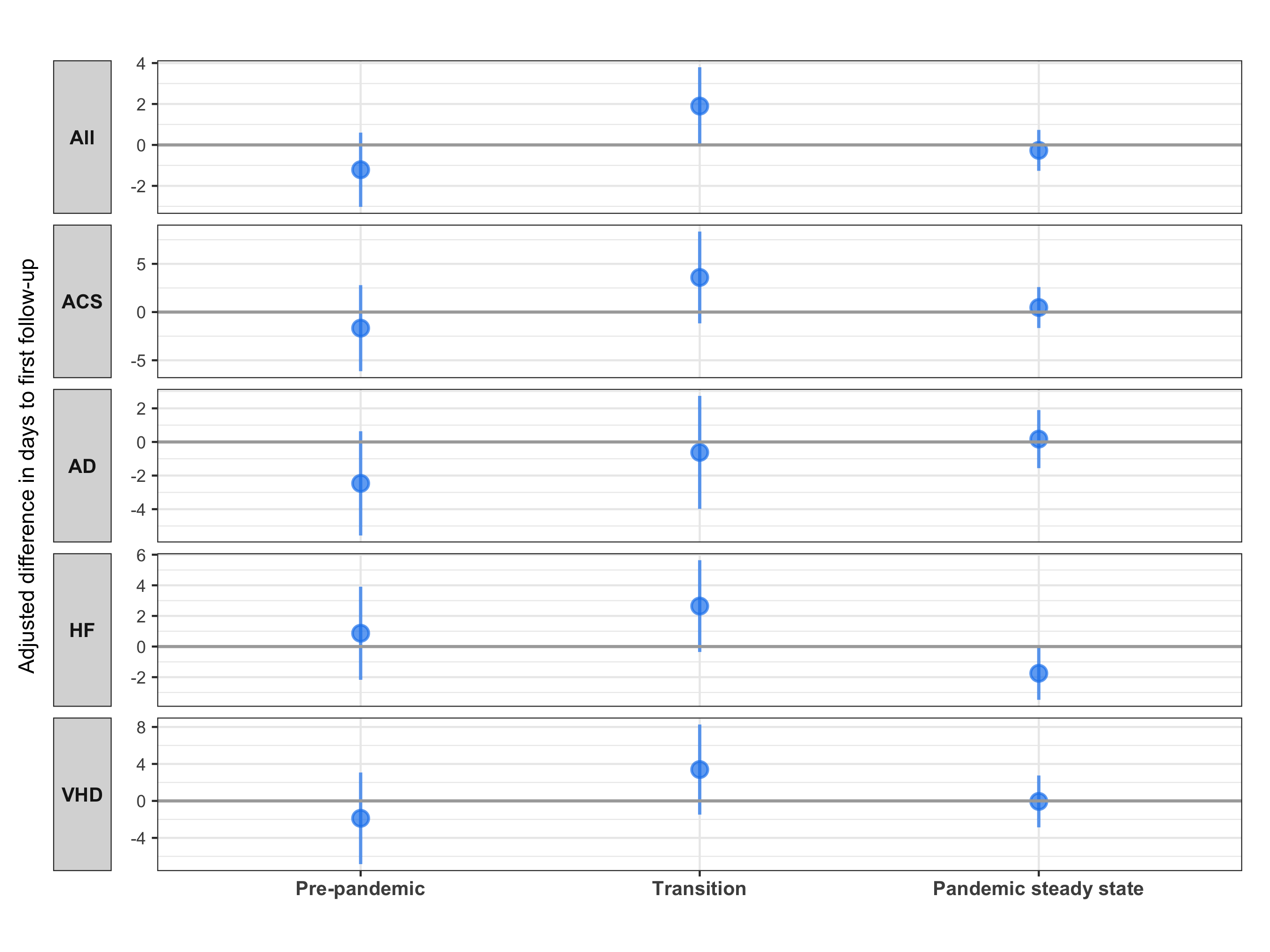


**Notes**: Data reflects encounters that had at least one follow-up within 60 days of discharge. Point estimates represent differences in number of days to first follow-up in 2020 vs. 2019 in each of our three time periods. Estimates are adjusted for encounter and patient characteristics. Error bars reflect 95% confidence intervals with heteroskedasticity robust standard errors. ACS = acute coronary syndrome; AD = arrhythmia disorders; HF = heart failure; VHD = valvular heart disease.

**Supplemental Figure 7.** Unadjusted 30-day all-cause unplanned readmission rates during the pre-pandemic, transition, and pandemic steady state time periods


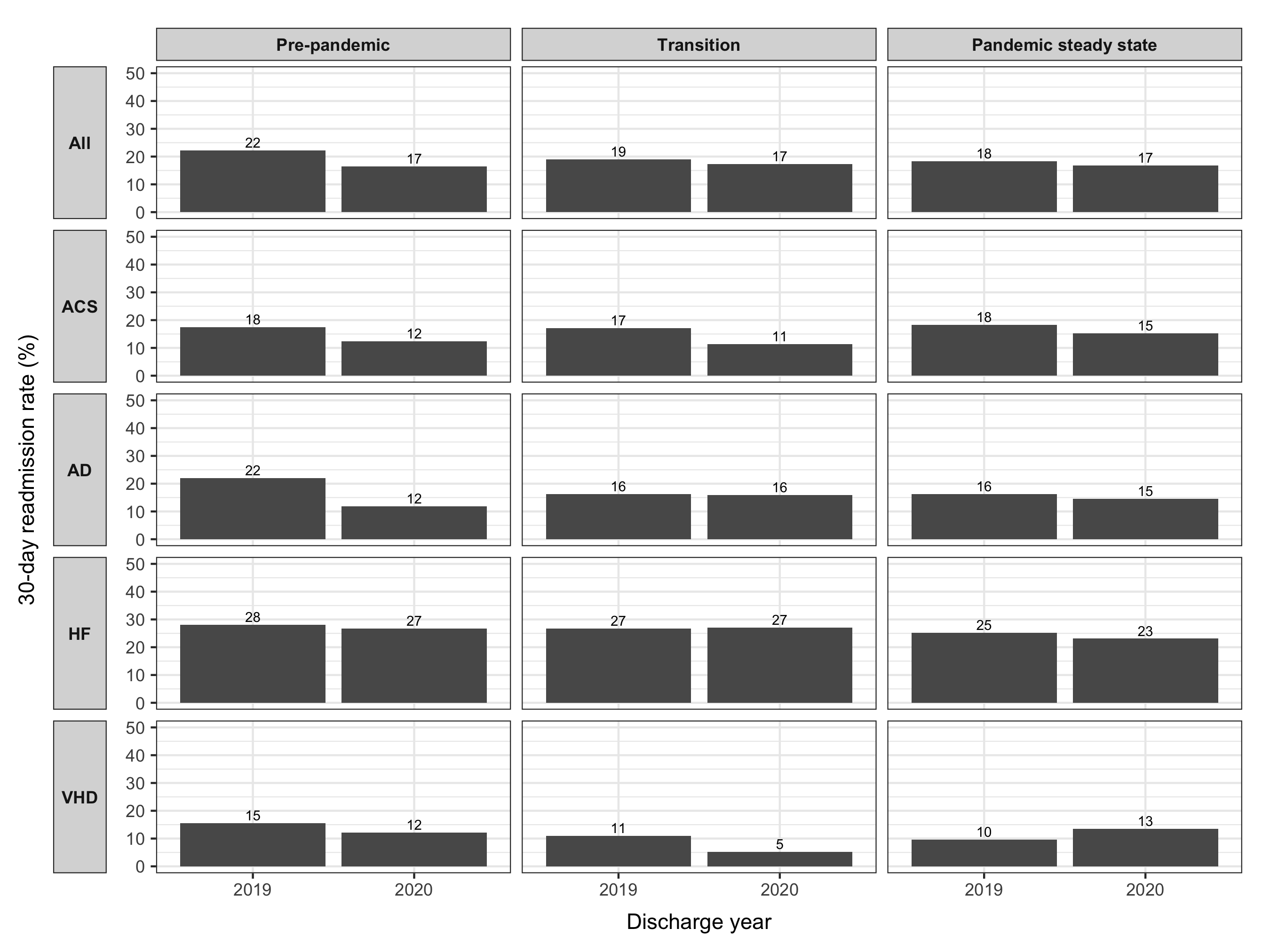


**Notes:** 30-day unplanned readmissions include any emergency department, observation, or inpatient stays within 30 days of the index encounter discharge date. Readmissions are not restricted to those with cardiology primary diagnosis codes. ACS = acute coronary syndrome; AD = arrhythmia disorders; HF = heart failure; VHD = valvular heart disease.

**Supplemental Figure 8.** 2020 vs. 2019 adjusted difference in 30-day all-cause unplanned readmission rate by month


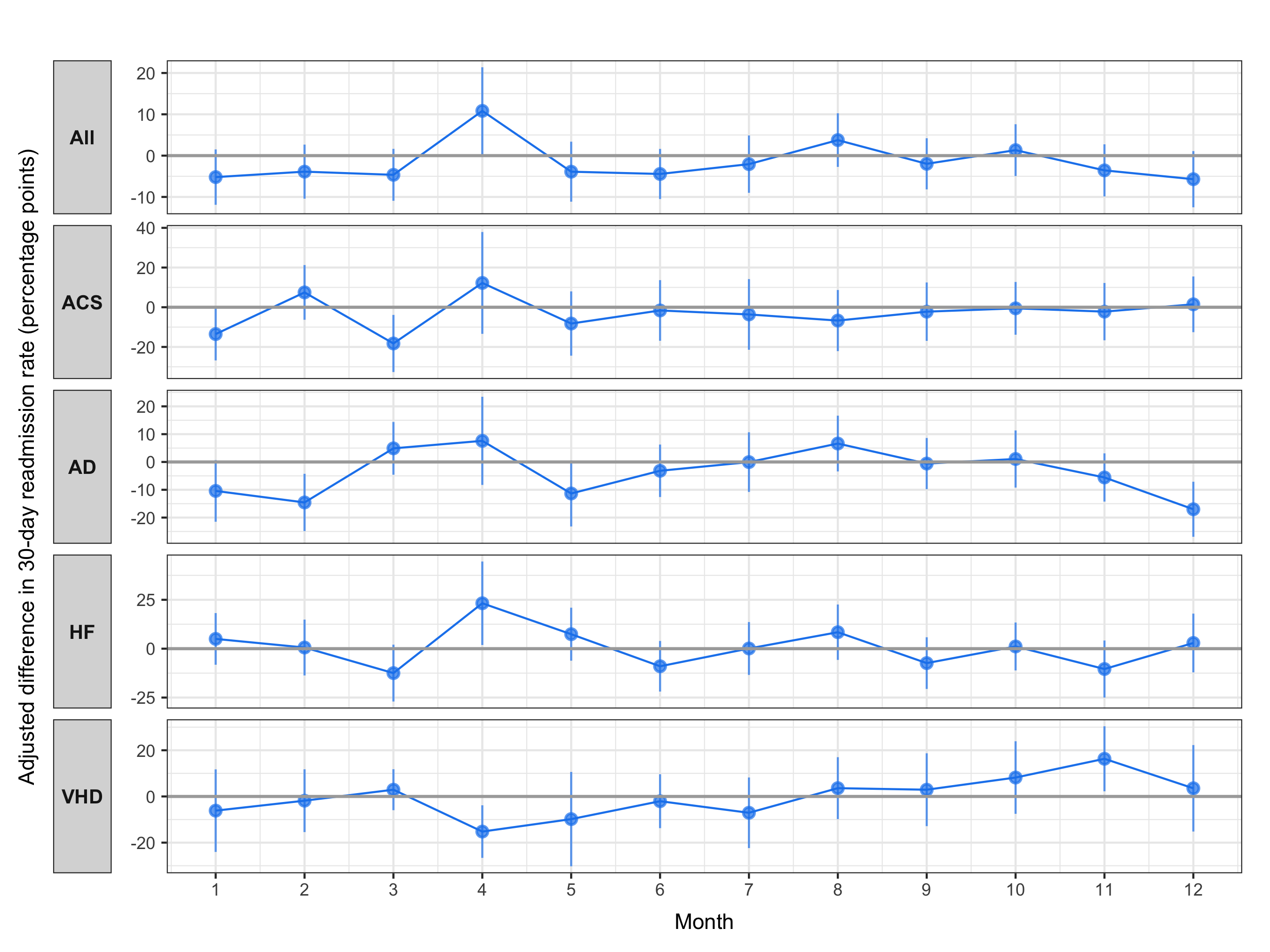


**Notes**: Point estimates represent differences in readmission rates in 2020 vs. 2019 in each month. Estimates are adjusted for encounter and patient characteristics. Error bars reflect 95% confidence intervals with heteroskedasticity robust standard errors. ACS = acute coronary syndrome; AD = arrhythmia disorders; HF = heart failure; VHD = valvular heart disease.

**Supplemental Table 4.** 2019 follow-up characteristics by cardiovascular condition

| **Condition** | **# of encounters** | **Time to follow-up (days)** | **14-day follow-up rate** | **28-day follow-up rate** | **Follow-up booked during hospitalization** | **Time to follow-up (days) –**  **booked during hospitalization** | **Follow-up with cardiology** | **Follow-up with physician** |
| --- | --- | --- | --- | --- | --- | --- | --- | --- |
| ACS | 487 | 17.1 | 54.8% | 79.3% | 20.9% | 16.0 | 71.5% | 67.4% |
| AD | 938 | 17.8 | 53.5% | 76.7% | 16.8% | 14.7 | 69.2% | 70.1% |
| HF | 834 | 14.1 | 65.2% | 86.9% | 30.6% | 9.2 | 64.6% | 58.3% |
| VHD | 416 | 23.4 | 33.9% | 59.9% | 19.2% | 24.5 | 78.4% | 67.3% |
| All | 2675 | 17.4 | 54.4% | 77.7% | 22.2% | 13.9 | 69.6% | 65.5% |

**Notes**: Measures are provided for the first follow-ups for all in-scope encounters in 2019 with ≥1 follow-up within 60 days of discharge. A follow-up was considered to be “booked during hospitalization” if the date on which the follow-up was booked was on or after the admit date and on or before the discharge date. A follow-up was considered “with cardiology” if the patient saw either a cardiology or cardiac surgery physician or advance practice provider. ACS = acute coronary syndrome; AD = arrhythmia disorders; HF = heart failure; VHD = valvular heart disease.
